# Association of sex and age with in‐hospital mortality and complications of patients with intracerebral hemorrhage: A study from the Chinese Stroke Center Alliance

**DOI:** 10.1002/brb3.2846

**Published:** 2022-12-10

**Authors:** Ping Lu, Zhentang Cao, Hongqiu Gu, Zixiao Li, Yu Wang, Lingyun Cui, Yongjun Wang, Xingquan Zhao

**Affiliations:** ^1^ Department of Neurology Beijing Tiantan Hospital Capital Medical University Beijing People's Republic of China; ^2^ China National Clinical Research Center for Neurological Diseases Capital Medical University Beijing People's Republic of China; ^3^ Research Unit of Artificial Intelligence in Cerebrovascular Disease Chinese Academy of Medical Sciences Beijing People's Republic of China; ^4^ Department of Neurology First Affiliated Hospital of Zhengzhou University Zhengzhou People's Republic of China

**Keywords:** age, intracerebral hemorrhage, mortality, sex

## Abstract

**Background and purpose:**

The impact of sex and age on prognosis in patients with intracerebral hemorrhage (ICH) in the Chinese population remains unclear. Our study aimed to investigate the relationship between sex and age of Chinese ICH patients and adverse prognosis.

**Methods:**

We used the Chinese Stroke Center Alliance database with in‐hospital mortality as the primary outcome and hospital complications as the secondary outcome. Patients were divided into four groups by sex and age. Logistic regression analyses were performed to assess the association between sex and age and the prognosis of ICH patients.

**Results:**

We enrolled 60,911 ICH patients, including 22,284 young and middle‐aged males, 15,651 older males, 11,948 young and middle‐aged females, and 11,028 older females. After adjusting for variables, older male patients had a higher mortality rate (OR = 1.21, 95% CI 1.01–1.45), combined with more frequent hematoma expansion (OR = 1.14, 95% CI 1.03–1.26), pneumonia (OR = 1.91, 95% CI 1.81–2.03), and hydrocephalus (OR = 1.28, 95% CI 1.04–1.59). Young and middle‐aged female patients had a lower mortality rate (OR = 0.74, 95% CI 0.58–0.95) and less frequent combined pneumonia (OR = 0.81, 95% CI 0.75–0.87). In‐hospital mortality was not significantly different in older females compared with young and middle‐aged males, but the odds of deep vein thrombosis, swallowing disorders, urinary tract infections, and gastrointestinal bleeding were significantly higher.

**Conclusion:**

Among young and middle‐aged patients, females are related to a lower in‐hospital mortality rate from ICH. Older patients are at an increased risk of ICH complications, with higher in‐hospital mortality in older men.

## Graphical abstract

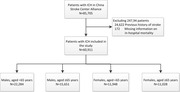

## 1 INTRODUCTION

Ten percent to 15% of strokes are intracerebral hemorrhage (ICH), which has an in‐hospital mortality rate of 30–40% (Rincon & Mayer et al., 2013; van Asch et al., 2010), with hematoma expansion and complications in patients with early ICH being the leading cause of death (Dowlatshahi et al., 2011; Hansen et al., 2013).

There are significant differences between males and females in the occurrence, presentation, mechanisms, treatment, and prognosis of cardiovascular and cerebrovascular diseases (Bushnell et al., 2014; Di Carlo et al., 2003; Regitz‐Zagrosek & Kararigas et al., 2017; Seshadri & Wolf et al., 2007). Management of ICH needs to be based on sex differences to avoid misunderstandings and bias (The Lancet Network, 2019). There are previous contradictions regarding sex differences and mortality after ICH (Ayala et al., 2002; Bushnell et al., 2014; Carcel et al., 2019; Ganti et al., 2013; Nilsson et al., 2002; Purroy et al., 2019; Shigematsu et al., 2015; Sheikh & Bullock et al., 2007; Umeano et al., 2013; Zia et al., 2009). This may be due to the fact that aging affects the release of systemic substances in people of the same sex, affecting clinical recovery after ICH (Engman et al., 2012; Guevara et al., 2011; Hsieh et al., 2016; Lei et al., 2016). In the face of global aging, analyzing the impact of different sex and age on the treatment and prognosis of ICH would be beneficial for the development of treatment strategies and to reduce the global health burden (Cao et al., 2020; Feigin et al., 2017).

In this study, we analyzed all patients with ICH who met the inclusion criteria in the Chinese Stroke Center Alliance (CSCA) study with the aim of investigating the impact of sex and age on the adverse prognosis of ICH patients. Through this study, we aim to promote the study of sex and age differences in ICH and to fill research gaps to improve the diagnosis and management of this disease.

## 2 METHODS

### 2.1 Study cohort and participants

This study was based on data from the CSCA (Wang et al., 2018). The CSCA is a multicenter registry study that enrolled Chinese patients aged ≥18 years with ischemic stroke, transient ischemic attack, primary ICH (excluding traumatic ICH), and subarachnoid hemorrhage who were seen for hospitalization within 7 days of the cerebrovascular event. The CSCA study was initiated in August 2015 and was completed in July 2019. A total of 1476 sentinel hospitals participated in the study, enrolling 85,705 ICH patients (Gu et al., 2021). The study was conducted in accordance with the Declaration of Helsinki and was approved by the Ethics Committee. All patients with a preliminary diagnosis of ICH were included, with the following exclusion criteria: (van Asch et al., 2010) previous stroke history; (Rincon & Mayer, 2013) missing data on sex and age; (Dowlatshahi et al., 2011) missing data on in‐hospital mortality.

### 2.2 Patient characteristics

Data on patient demographics and clinical characteristics (degree of neurological impairment, medical history, behavioral history, medication history, laboratory tests, prognostic information, and in‐hospital interventions) were collected and managed based on a web‐based system. In‐hospital data were recorded by the CSCA, and patients were not followed up after discharge.

### 2.3 Subgroup methods

Based on the World Health Organization's classification of population age, we considered patients aged ≥65 years as an older population (The Lancet Network, 1995). According to sex and age, patients were divided into four groups: young and middle‐aged males (< 65 years); older males (≥65 years); young and middle‐aged females (< 65 years); and older females (≥65 years). The reference group was the young and middle‐aged male group.

### 2.4 Outcome

The primary outcome of interest was the adverse prognosis during hospitalization, including mortality, hematoma expansion, urinary tract infection, pneumonia, dysphagia, epilepsy, deep vein thrombosis, gastrointestinal bleeding, and hydrocephalus.

### 2.5 Sensitivity analysis

The Glasgow Coma Scale (GCS) and the National Institutes of Health Stroke Scale (NIHSS) are considered proxies to assess the severity of ICH. Due to the relatively high proportion of missing data for NIHSS scores and GCS scores (47.9% and 64.9%, respectively), no adjustment was made in the primary model for the outcome analysis. In the sensitivity analysis, NIHSS scores and GCS scores were included in the adjusted model.

### 2.6 Statistical analysis

Continuous variables are expressed as mean ± standard deviation or median (interquartile range), and categorical variables are expressed as frequencies and percentages. Continuous variables were compared between groups using analysis of variance or Wilcoxon's rank‐sum test, while Pearson's chi‐square test was used to compare categorical variables. Corrected odds ratios (ORs) with 95% confidence intervals (CIs) were calculated by multifactorial logistic regression to determine the association between sex/age and prognosis. Imbalances in the baseline analysis (*p* < .05) were used as adjusted variables. Two‐sided *p* values of less than .05 were considered statistically significant. Statistical analyses were performed using SAS software V.9.4 (Gu et al., 2018).

## 3 RESULTS

### 3.1 Baseline characteristics

**FIGURE 1:**
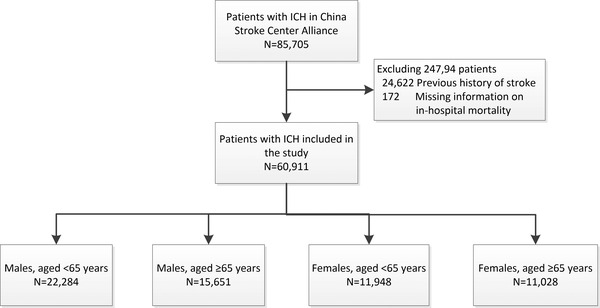
Study population flowchart

In total, 85,705 patients with ICH were enrolled in the CSCA between August 2015 and July 2019. There were no missing data on sex and age, and after excluding patients with a previous history of stroke (*n* = 24,622) and missing data on in‐hospital death (*n* = 172), a total of 60,911 patients with ICH were enrolled (Figure 1).

Of these patients, 36.6% were young and middle‐aged males (mean age, 52.3 years), 25.7% were older males (mean age, 73.9 years), 19.6% were young and middle‐aged females (mean age, 53.7 years), and 18.1% were older females (mean age, 75.0 years). Smoking, alcohol consumption, hyperlipidemia, and elevated uric acid were more common in young and middle‐aged males, and myocardial infarction, peripheral vascular disease, and elevated homocysteine were more frequent in older males. Young and middle‐aged females had fewer vascular risk factors compared with other groups, whereas older females had more frequent hypertension, diabetes mellitus, atrial fibrillation, heart failure, and higher glycated hemoglobin, fasting blood glucose (FBG), low‐density lipoprotein, and stroke severity. Moreover, hematoma removal was more common in young and middle‐aged patients compared with older patients, especially in young and middle‐aged males (Table 1).

**TABLE 1:** Clinical characteristics by sex and age among ICH admissions

Variables	Q1 (N = 22,284)	Q2 (N = 15,651)	Q3 (N = 11,948)	Q4 (N = 11,028)	p value
**Clinical features, mean ± SD/n (%)**					
SBP (mmHg)	165.2 ± 28.8	164.8 ± 27.0	165.1 ± 29.2	167.1 ± 28.5	<.001
DBP (mmHg)	99.6 ± 17.8	92.3 ± 15.4	96.3 ± 16.7	91.4 ± 16.1	<.001
Current smoking	8415 (37.8)	4090 (26.1)	179 (1.5)	264 (2.4)	<.001
Drinking	9610 (43.1)	5030 (32.1)	316 (2.6)	289 (2.6)	<.001
**Medical history, n (%)**					
Hypertension	14,675 (65.9)	10,170 (65.0)	8471 (70.9)	7678 (69.6)	<.001
Diabetes mellitus	1565 (7.0)	1221 (7.8)	928 (7.8)	1203 (10.9)	<.001
Dyslipidemia	675 (3.0)	349 (2.2)	334 (2.8)	270 (2.4)	<.001
Atrial fibrillation	105 (0.5)	289 (1.8)	45 (0.4)	259 (2.3)	<.001
Heart failure	47 (0.2)	82 (0.5)	20 (0.2)	83 (0.8)	<.001
Myocardial infarction	125 (0.6)	178 (1.1)	35 (0.3)	100 (0.9)	<.001
Peripheral vascular disease	82 (0.4)	102 (0.7)	44 (0.4)	62 (0.6)	<.001
**Baseline medication, n (%)**					
Antiplatelet	517 (2.3)	636 (4.1)	259 (2.2)	425 (3.9)	<.001
Anticoagulation	222 (1.0)	210 (1.3)	114 (1.0)	147 (1.3)	<.001
Antidiabetic	1063 (4.8)	893 (5.7)	645 (5.4)	878 (8.0)	<.001
Cholesterol‐reduce	268 (1.2)	264 (1.7)	106 (0.9)	190 (1.7)	<.001
**Lab tests, mean ± SD**					
FBG	6.4 ± 2.7	6.3 ± 2.5	6.6 ± 2.9	6.8 ± 2.9	<.001
Glycosylated hemoglobin	5.8 ± 1.6	5.8 ± 1.5	5.9 ± 1.7	6.0 ± 1.7	<.001
Platelet count	207.1 ± 68.8	187.0 ± 66.0	219.2 ± 72.7	202.2 ± 70.2	<.001
LDL	2.8 ± 1.5	2.6 ± 1.4	2.9 ± 1.5	2.9 ± 1.5	<.001
Homocysteine	17.8 ± 14.8	18.2 ± 14.6	14.0 ± 12.2	15.5 ± 12.7	<.001
Creatinine	142.1 ± 1271.4	135.3 ± 1208.2	135.6 ± 1666.8	111.8 ± 929.1	<.001
BUN	5.6 ± 2.8	6.0 ± 2.7	5.2 ± 2.6	5.8 ± 2.7	<.001
Uric acid	320.5 ± 141.0	300.2 ± 122.6	250.1 ± 122.8	255.4 ± 120.3	<.001
NIHSS, mean ± SD	7.5 ± 8.2	8.2 ± 8.5	8.2 ± 8.7	9.0 ± 8.8	<.001
GCS, mean ± SD	11.8 ± 4.1	11.7 ± 4.0	11.5 ± 4.1	11.3 ± 4.1	<.001
Prestroke mRS, n (%)					<.001
0–2	17,114 (76.8)	11,654 (74.5)	9267 (77.6)	8185 (74.2)	
3–5	5168 (23.2)	3995 (25.5)	2679 (22.4)	2841 (25.8)	
LOS, mean ± SD	16.8 ± 12.0	16.0 ± 11.3	17.5 ± 11.4	16.0 ± 11.3	<.001
Hematoma evacuation, n (%)	2840 (12.7)	1260 (8.1)	1500 (12.6)	864 (7.8)	<.001

Abbreviations: BUN, blood urea nitrogen; DBP, diastolic blood pressure; FBG, fasting blood glucose; GCS, Glasgow Coma Scale; ICH, intracerebral hemorrhage; LDL, low‐density lipoprotein; LOS, length of stay; mRS, modified Rankin Scale; NIHSS, National Institutes of Health Stroke Scale; SBP, systolic blood pressure.

### 3.2 Adverse prognostic outcome

Overall, 1307 (2.1%) ICH patients died during hospitalization, including 2.0% of young and middle‐aged males, 2.6% of older males, 1.4% of young and middle‐aged females, and 2.6% of older females. Diastolic blood pressure, systolic blood pressure, smoking, alcohol consumption, hypertension, diabetes mellitus, hyperlipidemia, atrial fibrillation, heart failure, myocardial infarction, peripheral vascular disease, antiplatelet agents, anticoagulants, hypoglycemic agents, lipid‐lowering agents, FBG, glycated hemoglobin, platelets, low‐density lipoprotein, homocysteine, creatinine, blood urea nitrogen, uric acid, prestroke modified Rankin Scale, length of hospitalization, and hematoma removal were adjusted in regression. The adjusted ratio of in‐hospital mortality was lower in young and middle‐aged females (OR = 0.74, 95% CI 0.58‐0.95, *p* = .016) and higher in older males (OR = 1.21, 95% CI 1.01‐1.45, *p* = .036) compared with young and middle‐aged males; however, no significant difference was found among older female patients (OR = 1.08, 95% CI 0.87‐1.34, *p* = .493) (Tables 2 and 3).

**TABLE 2:** ORs (95% CI) for in‐hospital mortality and complications in patients with ICH according to sex and age

		Odds ratio(95% Cl)				Sensitivity analysis^a^	
Outcomes	Events n(%)	Unadjusted OR(95% Cl)	p value	Adjusted OR(95% Cl)	p value	Adjusted OR(95% Cl)	p value
**In‐hospital mortality**							
Male, age < 65 years	440 (1.97)	Reference		Reference		Reference	
Male, age≥65 years	412 (2.63)	1.34 (1.17‐1.54)	<.001	1.21 (1.01‐1.45)	.036	1.16 (0.90‐1.48)	.251
Female, age < 65 years	164 (1.37)	0.69 (0.58‐0.83)	<.001	0.74 (0.58‐0.95)	.016	1.00 (0.74‐1.35)	.985
Female, age≥65 years	291 (2.64)	1.35 (1.16‐1.56)	.001	1.08 (0.87‐1.34)	.493	0.80 (0.58‐1.11)	.182
**Hematoma expansion**							
Male, age < 65 years	1424 (6.39)	Reference		Reference		Reference	
Male, age≥65 years	989 (6.32)	0.99 (0.91 ‐1.08)	.796	1.14 (1.03‐1.26)	.013	1.16 (0.90‐1.48)	.251
Female, age < 65 years	719 (6.02)	0.94 (0.85‐1.03)	.170	1.07 (0.95‐1.20)	.287	1.00 (0.74‐1.35)	.985
Female, age≥65 years	643 (5.83)	0.91 (0.82‐1.00)	.045	1.09 (0.96‐1.23)	.200	0.80 (0.58‐1.11)	.182
**Pneumonia**							
Male, age < 65 years	4771 (21.41)	Reference		Reference		Reference	
Male, age≥65 years	4842 (30.94)	1.65 (1.57‐1.73)	<.001	1.91 (1.81‐2.03)	<.001	2.06 (1.81‐2.33)	<.001
Female, age < 65 years	2176 (18.21)	0.82 (0.77‐0.86)	<.001	0.81 (0.75‐0.87)	<.001	0.82 (0.69‐0.96)	.016
Female, age≥65 years	3066 (27.80)	1.41 (1.34‐1.49)	<.001	1.59 (1.48‐1.71)	<.001	1.70 (1.45‐1.98)	<.001
**Poor swallow function**							
Male, age < 65 years	2826 (18.04)	Reference		Reference		Reference	
Male, age≥65 years	2676 (23.51)	1.40 (1.32‐1.48)	<.001	1.46 (1.36‐1.58)	<.001	1.53 (1.31‐1.79)	<.001
Female, age < 65 years	1502 (17.66)	0.98 (0.91‐1.04)	.469	0.94 (0.86‐1.03)	.190	0.81 (0.67‐1.00)	.046
Female, age≥65 years	2067 (26.23)	1.62 (1.52‐1.72)	<.001	1.69 (1.55‐1.85)	<.001	1.78 (1.47‐2.14)	<.001
**Seizure**							
Male, age < 65 years	255 (1.14)	Reference		Reference		Reference	
Male, age≥65 years	179 (1.14)	1.00 (0.83 ‐1.21)	.998	1.13 (0.89‐1.44)	.307	1.70 (1.06‐2.74)	.029
Female, age < 65 years	119 (1.00)	0.87 (0.70‐1.08)	.206	1.08 (0.81‐1.44)	.603	1.16 (0.63‐2.14)	.639
Female, age≥65 years	105 (0.95)	0.83 (0.66‐1.04)	.110	0.97 (0.71‐1.33)	.859	1.34 (0.74‐2.44)	.341
**Urinary tract infection**							
Male, age < 65 years	364 (1.63)	Reference		Reference		Reference	
Male, age≥65 years	326 (2.08)	1.28 (1.10‐1.49)	.001	1.31 (1.09‐1.57)	.004	1.32 (0.90‐1.93)	.161
Female, age < 65 years	337 (2.82)	1.75 (1.50‐2.03)	<.001	1.70 (1.40‐2.05)	<.001	2.20 (1.46‐3.32)	<.001
Female, age≥65 years	353 (3.20)	1.99 (1.72‐2.31)	<.001	2.16 (1.78‐2.61)	<.001	2.29 (1.51‐3.49)	<.001
**Hydrocephalus**							
Male, age < 65 years	418 (1.88)	Reference		Reference		Reference	
Male, age≥65 years	307 (1.96)	1.05 (0.90‐1.22)	.541	1.29 (1.08‐1.55)	.005	1.34 (0.89‐2.01)	.167
Female, age < 65 years	250 (2.09)	1.12 (0.95‐1.31)	.170	1.24 (1.01‐1.53)	.037	1.18 (0.73‐1.90)	.491
Female, age≥65 years	212 (1.92)	1.03 (0.87‐1.21)	.773	1.28 (1.04‐1.59)	.023	1.09 (0.66‐1.80)	.739
**Deep vein thrombosis**							
Male, age < 65 years	205 (0.92)	Reference		Reference		Reference	
Male, age≥65 years	209 (1.34)	1.46 (1.20‐1.77)	<.001	1.59 (1.27‐2.00)	<.001	1.41 (0.83‐2.40)	.203
Female, age < 65 years	126 (1.05)	1.15 (0.92‐1.43)	.228	1.32 (1.00 1.74)	.048	2.09 (1.14‐3.84)	.017
Female, age≥65 years	202 (1.83)	2.01 (1.65‐2.44)	<.001	2.37 (1.83‐3.06)	<.001	2.07 (1.11‐3.85)	.022
**Gastrointestinal bleeding**							
Male, age < 65 years	550 (2.47)	Reference		Reference		Reference	
Male, age≥65 years	449 (2.87)	1.17 (1.03‐1.33)	.016	1.22 (1.04‐1.42)	.014	0.94 (0.69‐1.30)	.722
Female, age < 65 years	225 (1.88)	0.76 (0.65‐0.89)	<.001	0.86 (0.71‐1.05)	.146	0.50 (0.32‐0.78)	.002
Female, age≥65 years	328 (2.97)	1.21 (1.05‐1.39)	.007	1.36 (1.123‐1.63)	.001	0.91 (0.62‐1.33)	.616

*Note*: Diastolic blood pressure, systolic blood pressure, smoking, alcohol consumption, hypertension, diabetes mellitus, hyperlipidemia, atrial fibrillation, heart failure, myocardial infarction, peripheral vascular disease, antiplatelet agents, anticoagulants, hypoglycemic agents, lipid‐lowering agents, fasting blood glucose, glycated hemoglobin, platelets, low‐density lipoprotein, homocysteine, creatinine, blood urea nitrogen, uric acid, prestroke modified Rankin Scale, length of hospitalization, and hematoma removal were adjusted in regression.

Abbreviation: ICH, intracerebral hemorrhage.

^a^ NIHSS scores and GCS scores were included in the adjusted model.

**TABLE 3:** Effect of sex differences on in‐hospital mortality in different age groups with ICH

	Death patients, n (%)		Odds ratio(95% Cl)						Sensitivity analysis^a^		
Group	Male	Female	Unadjusted OR(95%)	p value	Interaction p	Adjusted OR(95%)	p value	Interaction p	Adjusted OR(95%)	p value	Interaction p
Age < 65 years	440 (1.97)	164 (1.37)	0.69 (0.58‐0.83)	<.001	.002	0.79 (0.61‐1.02)	.071	.234	1.29 (0.67‐2.49)	.450	.822
Age≥65 years	412 (2.63)	291 (2.64)	1.00 (0.86‐1.17)	.975		0.84 (0.68‐1.04)	.108		0.90 (0.53‐1.51)	.677	

*Note*: Diastolic blood pressure, systolic blood pressure, smoking, alcohol consumption, hypertension, diabetes mellitus, hyperlipidemia, atrial fibrillation, heart failure, myocardial infarction, peripheral vascular disease, antiplatelet agents, anticoagulants, hypoglycemic agents, lipid‐lowering agents, fasting blood glucose, glycated hemoglobin, platelets, low‐density lipoprotein, homocysteine, creatinine, blood urea nitrogen, uric acid, prestroke modified Rankin Scale, length of hospitalization, and hematoma removal were adjusted in regression.

Abbreviation: ICH, intracerebral hemorrhage.

^a^ NIHSS scores and GCS scores were included in the adjusted model.

Young and middle‐aged females were related with a lower incidence of pneumonia (OR = 0.81, 95% CI 0.75‐0.87, *p* < .001) and a higher incidence of urinary tract infection (OR = 1.70, 95% CI 1.40‐2.05, *p* < .001), hydrocephalus (OR = 1.24, 95% CI 1.01‐1.53, *p* < .037), and deep vein thrombosis (OR = 1.32, 95% CI 1.00‐1.74, *p* < .048). Older male patients were associated with increased odds of multiple in‐hospital complications, including hematoma expansion (OR = 1.14, 95% CI 1.03‐1.26, *p* = .013), pneumonia (OR = 1.91, 95% CI 1.81‐2.03, *p* < .001), dysphagia (OR = 1.46, 95% CI 1.36‐1.58, *p* < .001), urinary tract infection (OR = 1.31, 95% CI 1.09‐1.57, *p* < .004), deep vein thrombosis (OR = 1.59, 95% CI 1.27‐2.00, *p* < .001), and gastrointestinal bleeding (OR = 1.22, 95% CI 1.04‐1.42, *p* < .014). Older female patients were associated with higher rates of urinary tract infection (OR = 2.16, 95% CI 1.78‐2.61, *p* < .001), pneumonia (OR = 1.59, 95% CI 1.48‐1.71, *p* < .001), dysphagia (OR 1.69, 95% CI 1.55‐1.85, *p* < .001), deep vein thrombosis (OR = 2.37, 95% CI 1.83‐3.06, *p* < .001), gastrointestinal bleeding (OR = 1.36, 95% CI 1.13‐1.63, *p* < .001), and hydrocephalus (OR = 1.28, 95% CI 1.04‐1.59, *p* < .023) (Table 2).

### 3.3 Sensitivity analysis

A sensitivity analysis was performed by including NIHSS scores and GCS scores in the adjusted model. Adjusted sex and age differences were not significantly associated with in‐hospital mortality, hematoma expansion, epilepsy, hydrocephalus, or gastrointestinal bleeding; however, significant associations were found with pneumonia, dysphagia, urinary tract infection, and deep vein thrombosis, with similar trends to the formal adjusted model.

## 4 DISCUSSION

Our study concluded that in ICH patients, young and middle‐aged females were related to lower in‐hospital mortality, while older males were related to an increased risk of in‐hospital mortality. Overall, older patients were at an increased risk of ICH‐related complications.

### 4.1 Sex differences and mortality

We speculate that loss of gonadal hormones after menopause may contribute to the sex difference in in‐hospital mortality in patients of different ages. Animal experiments have shown that female gonadal steroids reduce early brain edema and neuroinflammation, have neuroprotective effects, and promote brain recovery after craniocerebral trauma (Hsieh et al., 2016; Lei et al., 2016).In human studies, the cerebrovascular response is strongest in premenopausal females and less strong in postmenopausal females compared with males. Moreover, there is no difference between postmenopausal and premenopausal females in terms of the cerebrovascular response after estrogen replacement therapy (Matteis et al., 1998). Meanwhile, dysregulation of the gut microbiota after ICH contributes to the progression of neuroinflammation‐related brain injury (Yu et al., 2021), and sex‐related differences in the gut microbiota may be associated with gonadal steroids (Ahmed & Spence et al., 2021), which may lead to different degrees of inflammation.

### 4.2 Age differences and mortality

The higher in‐hospital mortality rate after ICH in older patients may be related to pathogenesis, hematoma volume, antithrombotic decision‐making, and neuroinflammation. The increased proportion of ICH cases caused by amyloid angiopathy and antithrombotic drug use in older patients and the higher incidence of lobar hemorrhage (Bejot et al., 2013) may lead to a higher risk of hematoma enlargement, which is consistent with our findings. This may account for the increase in hematoma volume with age (Kuramatsu et al., 2011). Also, the use of antithrombotic drugs after concomitant ICH in diseases, such as valvular heart disease and atrial fibrillation, remains controversial and may increase the risk of hemorrhage. The inflammatory response in older patients is complex, and the poorer prognosis of older patients may be associated with macrophage/microglia activation (Lively & Schlichter et al., 2012). In terms of clinical interventions, this study found that young and middle‐aged patients were more aggressively selected for hematoma removal, possibly explaining the lower mortality rate in young and middle‐aged patients. No sex differences were found, which is consistent with a previous study (Guha et al., 2017). In this study, the in‐hospital mortality rate for patients with ICH was 2.1%, much lower than the 30‐40% seen in previous studies. This may be related to the Chinese government's efforts to promote public education and primary stroke prevention, the age‐standardized mortality rate for patients with ICH decreased by 48.1% compared to the previous (Wang et al., 2022). At the same time, the data set is mainly from second‐ and third‐level hospitals, which may underestimate the mortality of ICH patients.

### 4.3 Sex, age, and complications

The risk of hematoma expansion may be related to sex. Previous studies have shown that females are less likely to demonstrate low serum cholesterol than males (Winston et al., 2009). Low serum cholesterol may affect the fragility and permeability of the vessel wall, and a protective effect may exist with higher low density lipoprotein (LDL) cholesterol levels (Bang et al., 2007). This is consistent with our study, where older male patients demonstrated lower LDL cholesterol levels with an increased risk of hematoma enlargement. Poststroke pneumonia significantly increases the risk of death (Katzan et al., 2003), and the incidence of pneumonia was significantly higher in older patients in our study, which may be related to the higher risk of dysphagia. Also, the incidence of stressful gastrointestinal bleeding was relatively high in older patients in our study. Acid‐suppressive agents are a common clinical strategy that may increase the risk of pulmonary infections with the long‐term application (Eom et al., 2011) and may not improve mortality. The higher incidence of urinary tract infections in females is well known and may be related to female physiology and a higher incidence of diabetes mellitus (Schneeberger et al., 2008). Relative to younger patients, older patients have an increased incidence of venous thromboembolism, more severe disease, and higher complications and mortality (Spirk et al., 2012), which may be associated with prolonged bed rest after ICH.

### 4.4 Sex and age in previous studies

In terms of age, previous studies have shown that mortality after ICH increases with age (20–49 years: 16.5%; ≥70 years: 23.9%) (Saqqur et al., 2020). The risk of in‐hospital death in patients with ICH ≥65 years old was increased in both men and women compared to < 65 years old (James et al., 2017). In terms of sex, some studies did not identify sex differences (Carcel et al., 2019) or age‐sex interaction effects (James et al., 2017) on mortality after ICH, while others found a higher mortality rate in females (Ganti et al., 2013; Nilsson et al., 2002; Purroy et al., 2019). In contrast, some studies have observed a lower mortality rate in females, especially in young and middle‐aged females after cross‐age grouping (Ayala et al., 2002; Bushnell et al., 2014; Olie et al., 2022; Sheikh & Bullock, 2007; Shigematsu et al., 2015; Umeano et al., 2013; Zia et al., 2009), which is similar to our findings. The reasons for these differences may be related to the different study types (retrospective vs. prospective), study populations (geographical vs. ethnic), inclusion criteria, analysis methods, and follow‐up evaluation methods. Sex and age differences were not significantly related to mortality in the sensitivity analysis. Instead, GCS and NIHSS scores at admission may be partially responsible for the differences in in‐hospital mortality.

### 4.5 Strengths and limitations

This study was a multicenter study, which attenuates geographical bias. Moreover, the sample size was large, and the data contained detailed clinical and demographic characteristics, which allowed for a more detailed assessment of ICH complications in addition to mortality. However, the present study has some limitations. First, this study did not have postdischarge follow‐up information to determine long‐term prognostic outcomes. Second, ICH imaging data were not collected to obtain information on hematoma volume and location. Third, a high percentage of NIHSS score and GCS score data were missing, and despite our sensitivity analysis, further discussion of these variables was a need.

## 5 CONCLUSION

Among patients with ICH, the female sex and age (young and middle‐aged) are protective factors for in‐hospital mortality, while older age and the male sex are risk factors for in‐hospital mortality. Overall, older patients had an increased risk of ICH‐related complications compared with young and middle‐aged patients. We conclude that sex and age are two major, unchangeable risk factors that should be considered in the development and clinical presentation of ICH. The social burden of ICH remains high, and new targeted therapies are needed to improve survival. Interventions for ICH, aging, and gonadal hormone levels merit further study.

## AUTHOR CONTRIBUTIONS

Ping Lu: study design, analysis and interpretation, and primary responsibility for writing the manuscript. Zhentang Cao, Hongqiu G, Zixiao Li, Yu Wang, Lingyun Cui, Yongjun Wang, and Xingquan Z: study design, data interpretation, critical revision of the manuscript for important intellectual content, and supervision of the study. Hongqiu G: data statistics. All authors contributed to the article and approved the submitted version.

## CONFLICT OF INTEREST

The research was conducted in the absence of any commercial or financial relationships that could be construed as a potential conflict of interest.

### PEER REVIEW

The peer review history for this article is available at https://publons.com/publon/10.1002/brb3.2846

## Data Availability

Data are available upon reasonable request. All data are available to researchers on request for purposes of reproducing the results or replicating the procedure by directly contacting the corresponding author.
